# Extensor Mechanism Reconstruction Using Allograft Following Total Knee Arthroplasty: A Review of Current Practice

**DOI:** 10.7759/cureus.12803

**Published:** 2021-01-20

**Authors:** Arnab Sain, Hemant Bansal, Kirubakaran Pattabiraman, Maximilian Muellner, Thomas Muellner

**Affiliations:** 1 Orthopaedics, All India Institute of Medical Sciences, New Delhi, IND; 2 Orthopaedics, Evangelisches Krankenhaus, Vienna, AUT

**Keywords:** allograft, extensor mechanism disruption, total knee arthroplasty, tka, extensor apparatus of the knee

## Abstract

The disruption of the extensor mechanism/apparatus of the knee is a dreaded complication following Total Knee Arthroplasty (TKA). Fresh frozen allograft containing the patella, and peripatellar musculotendinous attachments has emerged as an ideal alternative or salvageable option for the efficient reconstruction of extensor mechanism disrupted following TKA, where repair is almost impossible. However, any allograft implantation is associated with certain complications and extensor apparatus allograft is not the exception. Despite being allogenic, reconstruction of the extensor mechanism of the knee using allograft has given promising results. This narrative review aims to elaborate on the current application of allograft in the reconstruction of the disrupted extensor mechanism following TKA.

## Introduction and background

Disruption of the extensor mechanism of the knee is a rare and devastating complication after Total Knee Arthroplasty (TKA) which is difficult to treat and poses a great challenge to the restoration of the functional outcome of the knee [1-4]. The predisposing factors for disruption of extensor mechanism include obesity, chronic corticosteroid use, knee stiffness, pre-operative extensor mechanism complications, osteolysis, previous osteotomy, patella baja, and implant malposition [5].

Disruption of the extensor mechanism of the knee can occur from various causes like avulsion of the tibial tubercle, rupture of the patellar tendon, fracture of the patella, or rupture of quadriceps tendon [4]. Non-operative treatment is associated with poor functional outcome and operative management is the main treatment [6]. The primary repair has a high incidence of failure due to compromised blood supply along with damage or loss of soft tissue [4-7].

Many reconstruction procedures have been tried, such as soft tissue autograft, rotational muscle flaps, allograft, and synthetic graft materials, but the most promising results have been found with an allograft containing tibial tubercle, patellar tendon, patella, and quadriceps tendon. The allograft allows appropriate augmentation of damaged host tissue [6-8].

Emerson et al. reported the use of allograft to reconstruct the extensor mechanism of the knee [7,8]. Although the initial results were good, it was associated with extensor lag [7-9]. Nazarian and Booth suggested that the allograft should be tightly tensioned in full extension for better results [9].

Fresh, frozen allograft has higher strength, but it is more immunogenic than the freeze-dried allografts. Burnett et al. used fresh frozen allograft and found on histologic analysis that the graft incorporated well into the host tissue at all levels [10]. Also, the allograft has the advantage of avoiding the donor site morbidity associated with the use of autograft [4].

This article will focus on the use of the whole extensor mechanism allograft in the reconstruction of the disrupted extensor mechanism of the knee after TKA.

## Review

Materials and methods

A PubMed, Google Scholar, and Web of Science search was performed using the terms “Extensor Mechanism Allograft”, “Total Knee Arthroplasty”, and “Extensor mechanism reconstruction of the knee”. Only articles relating to extensor mechanism allograft reconstruction of disrupted extensor apparatus of the knee were selected for the study. Articles related to extensor mechanism allograft reconstruction in other pathologies of the knee and articles on other methods of extensor mechanism reconstruction of the knee were excluded from the study. The recently published articles were given more importance. The abstracts and the full text of the matched articles were collected and reviewed in detail. References that were cited in the identified articles were also screened for inclusion. The focus was to find out the current literature on this topic.

Indications of the procedure

Burnett et al. put forward the following indications for allograft reconstruction of the extensor mechanism of the knee [10]:

1. Disruption of the extensor mechanism which cannot be treated by primary repair or has a history of failed primary repair.

2. Prior excision, rupture, or avulsion of the patellar tendon.

3. Prior excision, rupture, or avulsion of quadriceps tendon.

4. Multifragmentary fracture of the patella where the patella cannot be reconstructed.

5. Extensive heterotopic ossification of the extensor mechanism of the knee.

6. Symptomatic extensor lag with the previous patellectomy with TKA.

7. Severe degree of patella baja which is causing restriction of movement.

8. Severe degree of arthrofibrosis of the knee.

9. TKA in cases of previous arthrodesis of the knee with a fibrosed/deficient extensor mechanism.

Contraindications of the procedure

The contraindications to the procedure as recommended by Burnett et al. [10] include:

1. Concurrent infection of the TKA.

2. Active infection at any local site near the concerned knee.

3. Extensor mechanism disruption where primary repair is possible.

4. Extensor mechanism disruption where reconstruction with autologous tissue graft is possible.

5. Non-compliant patient.

Pre-operative evaluation

A proper pre-operative evaluation is crucial to the success of the procedure. The pre-operative evaluation consists of taking a focussed history, relevant clinical examination, and necessary blood investigations and imaging [4,10].

*History Taking*: A detailed history of all the previous procedures on the affected knee should be sought. Any history of infection, medical co-morbidities, and any immune-suppressive therapy which might have an impact on wound healing should be investigated. Also, the patient should be asked about the current function of the knee joint and if there are any symptoms of instability while walking which might arise from extensor lag [4,10].

*Clinical Evaluation*: Clinical examination should focus on the presence of extensor lag and any palpable defect in the extensor mechanism of the knee. Also, the presence of any flexion and extension contracture and the full range of passive movement should be noted. Any mal-tracking of the patella should be checked as this is one of the main causes for rupture of the extensor mechanism after total knee arthroplasty. The soft tissue status of the knee should be evaluated and any need for soft tissue coverage procedures like flap coverage should be discussed with a plastic surgeon. The presence of previous incisions and any areas of decreased vascularity should be noted [4,10].

*Investigations*: Laboratory investigations like blood erythrocyte sedimentation rate (ESR), C-reactive protein, and biochemical and microbiological analysis of synovial fluid obtained by joint aspiration should aim at detecting any infection [4,10].

Imaging like radiographs and computed tomography of the knee joint should aim at detecting implant loosening, implant malrotation, and abnormal position of the patella which will require a revision of the implant before reconstruction of the extensor mechanism is done. Also, it can detect problems like heterotopic ossification, multi-fragmentary fracture of the patella, rupture/avulsion of the patella and quadriceps tendon, all of which may require allograft reconstruction [4,10].

Surgical technique

*Allograft*: The allograft used nowadays is a fresh frozen, non-irradiated specimen that contains the entire extensor mechanism of the same side as the affected knee is preferred. The use of allograft on the opposite side is not recommended. The use of fresh frozen allograft is preferred over the freeze-dried as Emerson et al. noted better results with the fresh frozen grafts and there were concerns that freeze-drying weakened the allograft leading to complications and graft failure [8]. The allograft should be checked pre-operatively to ensure that adequate specimen is available. The tibial bone stock should be a minimum of 5 cm with the patellar tendon attached to it and the length of the quadriceps tendon should have a minimum length of 5 cm [4,5,10,11]. Murgier et al. in their study used tibial bone stock of length 7 cm, width 15 mm, and depth 2 mm [12].

*Patient Positioning*: The patient is positioned supine on an operating table with a bump beneath the ipsilateral trochanter. A sterile pneumatic tourniquet can be used. The lower extremity is prepared and draped as far proximally as possible to allow adequate exposure of the proximal thigh [4,5,10].

*Operative Exposure*: Previous skin incisions should be noted and marked. Although a midline skin incision is preferred, previous skin incision should be used if present. If multiple skin incisions exist then the most lateral incision near the midline should be used to preserve the blood supply of the skin. In case of multiple surgeries with poor soft tissue, envelope advice should be sought from the plastic surgeon regarding the need for flap coverage or other soft tissue procedures [4,5,10].

After skin incision, the skin with subcutaneous flaps are raised and the retinaculum and extensor mechanism of the knee is exposed. A midline incision is given through the remnant of the extensor mechanism of the knee raising medial and lateral flaps of the retinaculum exposing the joint. If a remnant of the patella is present, it is osteotomized in the middle in line with the extensor retinaculum. The medial and lateral gutters and the supra-patellar pouch are recreated again. The midline incision is carried proximally into the quadriceps tendon and distally into the tibial tubercle with the elevation of soft tissue flaps on both sides [4,5,10].

*Revision of Total Knee Arthroplasty*: After exposure of the prosthetic joint, if any revision of implant is required, it is performed at this point. The TKA is always performed prior to insertion of the allograft. Flexion and extension gap balancing should be done. It should be noted that if a stemmed tibial implant is used, the host tibial tray should be prepared and fixation wires should be passed before insertion of the stemmed implant [4,5,10].

*Preparation of Allograft*: The allograft is then prepared on the back table by another team. An allograft with a tibial bone block of 5 cm length, 2 cm width, and 2 cm depth is preferred. The proximal portion of the bone block is dovetailed from the distal anterior to proximal posterior at a 30-45 degree angle to avoid proximal migration [4,5,10,11].

As per the procedure described by previous authors [4,5,10,11], the allograft should have at least 5 cm of quadriceps tendon proximally. The quadriceps tendon is prepared with two non-absorbable sutures running in a locked fashion on the medial and lateral aspect of the quadriceps tendon creating four strands of sutures. These four strands are used to pull the quadriceps tendon proximally with the knee in full extension during allograft fixation.

Murgier et al. [12] suggested a longitudinal split of the quadriceps tendon for ease of insertion and tensioning of the graft. Also, Murgier et al. suggested that the allograft should be soaked in a solution containing rifampicin ( 1200 mg in 1 litre of saline) for 20 minutes prior to preparation.

*Preparation of the Host Tibial Tray*: As per previous studies [4,5,10,11], a tibial tray is prepared with a minimum 15 mm distance from the tibial implant to prevent proximal migration of the allograft. The dimensions of the tibial tray should be just less than the size of the allograft tibial bone block (5 mm x 2 cm x 2 cm) to allow press-fit of the allograft in the tibial tray.

Murgier et al, however, used a different sized tibial tray which was just less than the tibial bone stock (7 cm x 1.5 cm x 2 cm).

*Fixation of the Tibial Side of Allograft*: The tibial bone stock is gently press-fit into the tibial tray with a bone tamp or punch the tibial side of the graft is secured by two to three stainless steel wires/fibre wires passed through drill holes in a medial to the lateral direction. The knots are secured on the lateral side as there is adequate soft tissue coverage possible on the lateral side. Additionally, a small fragment 4.5 mm compression screw can be passed in an anterior to the posterior direction to secure the tibial bone stock to the host tibial tray [4,5,10,11,12]. Murgier et al. used a patella-tibial cerclage wire tightened at 30 degrees of flexion to augment the allograft fixation.

*Tensioning and Suturing of the Quadriceps Tendon*: The four suture strands of the quadriceps tendon are used to pull the allograft quadriceps tendon. The host quadriceps tendon is pulled distally by non-absorbable Krackow sutures placed in medial and lateral sleeves of the distal quadriceps retinaculum. The knee is maintained in full extension throughout the procedure and the allograft is sutured in place beneath the host quadriceps tendon in a “vest over pants” fashion using number 5 non-absorbable sutures [4,5,10,11]. It should be noted that the graft should be tightly tensioned in full knee extension for clinical success as suggested by Nazarian and Booth [9]. Murgier et al., however, used a Pulvertaft weave repair of the two flaps of the host quadriceps tendon and allograft quadriceps [12].

*Closure of Wound*: The allograft is covered with the host retinaculum. Murgier et al. recommended making four anterior-posterior drill holes in the patella to allow fixation of retinaculum with the patella. Care is taken not to flex the knee intra-operatively and the wound is closed over a drain. A cast/splint is given to maintain full extension post-operatively. The drain is removed on the second postoperative day [4,5,10,11].

Post-operative care

The cast/splint to maintain extension is continued for six to eight weeks and then a Range of Motion (ROM) brace is given. Active flexion increment of 10 degrees every week is allowed till 12 weeks and then the restriction on flexion is lifted. Passive flexion is not allowed for the fear of stretching the graft and leading to extensor lag. Physiotherapy supervision is required for quadriceps stretching exercises and range of flexion exercises [4,5,10,11].

Reconstruction with Achilles tendon allograft

Another type of allograft used in the reconstruction of the extensor mechanism of the knee is the Achilles tendon allograft. It is used in cases where the patella is intact or the patellar component can be mobilized within 2-3 cm of the joint line. It can also be used in cases of rupture of quadriceps tendon with severe retraction and the whole extensor mechanism allograft is impossible to use in that situation [5,13,14,15,16].

Results

Emerson et al. first described the procedure of extensor mechanism reconstruction with allograft in disruption of extensor mechanism after TKA. They found better results with fresh frozen allografts [7,8]. Also, there was an incidence of extensor lag in three patients out of nine patients in follow-up [7,8]. Nazarian and Booth recommended tensioning the allograft in full extension as they found better results after allograft tensioning in extension [9]. Burnett et al. in their study compared the results between tensioned and Non-tensioned allograft extensor reconstruction and found better results with tensioned allografts [11].

The Knee Society Score (KSS) has been used by most recent studies in determining functional outcome after allograft reconstruction of the extensor mechanism of the knee. The summary of recent studies is as per Table 1.

**Table 1:** Functional outcome in terms of Knee Society Score (KSS) in recent studies using the allograft reconstruction of extensor mechanism after TKA TKA: Total Knee Arthroplasty

Study	Number of knees in study	Duration of follow-up	Pre-operative KSS	Post-operative KSS	Success
Burnett et al. [15] (2006)	19 knees	56 months	27	56	All 19 patients (100%)
Blanco et al. [16] (2013)	7 knees	25 months	26	82	All 7 patients (100%)
Brown et al. [17] (2015)	50 knees	68 months	33.9	75.9	31 out of 50 knees (62%)
Ricciardi et al. [6] (2016)	26 knees	57.6 years	101+/-38	116+/-40	18 out of 26 knees (69%)
Lamberti et al. [18] (2016)	7 knees	5.7 years	34+/-14.4	78.5+/-14.6	6 out of 7 knees (85.7 %)
Lim et al. [19] (2017)	16 knees	3.3 years	40+/-14	67+/-15	11 out of 16 patients (69%)

Regarding results of Achilles tendon allograft, Crossett et al. used Achilles tendon allograft with calcaneal bone graft for allograft reconstruction of the disrupted extensor mechanism of the knee after TKA with good short-term results [13]. Wise et al. used Achilles tendon allograft in the reconstruction of the failed extensor mechanism of the knee and obtained good long-term results [14].

Complications

The allograft reconstruction of the knee extensor mechanism is associated with a number of complications. Clinical failure was defined as KSS <60 and extensor lag of >30 which will need revision surgery [17].

*Extensor Lag*: It is the most common complication of allograft reconstruction of the extensor mechanism of the knee. Previously in studies by Leopold et al. [20], the high incidence of extensor lag of 59 degrees was due to lack of tensioning of the allograft. Nazarian and Booth recommended that the graft should be tightly tensioned in full knee extension for clinical success and they found a mean extensor lag of 13 degrees [9]. Burnett et al. in their study found that the extensor lag in the non-tensioned graft was 59 degrees compared to 4.3 degrees in tensioned graft [10,11]. Tendons are plastic structures and they elongate after healing, so the full extension is hard to achieve. By tensioning of allograft, the extensor lag can be reduced to a significant extent. Also, the graft is to be protected in a splint/cast for six to eight weeks following surgery.

*Rupture of the Quadriceps Tendon and Patellar Tendon of Allograft*: Rupture of the quadriceps tendon and patellar tendon of allograft as a complication was described by Emerson et al. [7,8] and Nazarian and Booth [9]. In the study by Emerson et al., the single case of quadriceps tendon rupture and one case of patellar tendon rupture were attributed to surgical damage during patellar resurfacing. In the study by Nazarian and Booth, out of the eight cases of failed reconstruction, six cases failed due to rupture of the quadriceps tendon. As there is a chance of surgical damage to the tendon and the patella of the allograft is insensate, patella resurfacing of the allograft is not recommended [7,8].

*Fracture of the Patella*: Patellar fracture of the allograft has been reported by Ricciardi et al. in their study [6] leading to the failure of the extensor mechanism of the knee.

*Failure of Fixation of Tibial Tubercle Bone Block*: The tibial tubercle bone fixation can fail due to poor fixation methods and due to non-compliance of the patient in the early postoperative period. Nazarian and Booth reported two cases of failure of the fixation of tibial tubercle bone block due to poor compliance with bracing mechanism [9].

*Infection*: Deep infection after allograft reconstruction was reported by Ricciardi et al. [6], Blanco et al. [16], and Brown et al. [17]. The use of allografts has been associated with infections. The infection is treated by debridement and antibiotics. In cases of failure of reconstruction due to infection, knee arthrodesis or amputation is done [6,16,17].

*Wound Complications*: Wound complications like necrosis of the wound were reported by Emerson et al. [7,8]. Pre-operative evaluation of soft tissues around the knee joint and involvement of a plastic surgeon for soft tissue procedures like flap coverage helps in reducing wound complications [4,5,10,11].

Survivorship of allograft

Regarding survival of allograft, young age is associated with decreased survival of graft and more complications. This may be due to poor compliance of the younger population with the post-operative knee immobilization protocol [6].

## Conclusions

Disruption of the extensor mechanism of the knee is a rare and devastating complication after Total knee Arthroplasty (TKA). Allograft reconstruction of the knee offers a reliable and effective solution in cases where primary repair is not possible. It avoids the disadvantages of morbidity associated with autografts. The fresh-frozen allograft is used as it is associated with fewer complications. Also, the graft should be tensioned in full extension of the knee to reduce the extent of extensor lag in the post-operative period. As it is an extremely rare procedure, the available studies have a very less number of subjects. More studies with a larger number of subjects are needed for determining the functional outcome of this procedure and the complications associated with this procedure.
